# Gestational Hypertension as a Mediator of Prenatal Ozone Exposure and Term Low Birth Weight: Birth Cohort Study

**DOI:** 10.2196/81412

**Published:** 2026-04-08

**Authors:** Yuxin Zhang, Quanjun Liu, Mengwei Song, Qiulin Huang, Qingbo Zhao, Qingqing Tao, Chenchen Zhang, Qin Li, Qing Wang

**Affiliations:** 1 Department of Biostatistics, School of Public Health, Cheeloo College of Medicine, Shandong University, No.44 Wenhuaxi Road, Jinan, 250012, China, 86 18811795046; 2 National Institute of Health Data Science of China, School of Public Health, Cheeloo College of Medicine, Shandong University, Jinan, China; 3 Department of Maternal and Child Health, School of Public Health, Peking University, Beijing, China; 4 NHC Key Lab of Health Economics and Policy Research, School of Public Health, Cheeloo College of Medicine, Shandong University, Jinan, China

**Keywords:** ozone exposure, low birth weight, small for gestational age, gestational hypertension, causal mediation

## Abstract

**Background:**

Ambient ozone (O_3_) exposure has been found to be associated with gestational hypertension, which, in turn, increases the risk of term low birth weight (LBW). As such, gestational hypertension acts as a potential mechanism mediating restricted fetal growth; however, few epidemiological studies have quantified this specific mediation pathway.

**Objective:**

This study aims to examine whether gestational hypertension serves as a mediator of the association between prenatal O_3_ exposure and term LBW.

**Methods:**

We conducted a population-based cohort study using the Cheeloo Lifespan Electronic Health Research Data-library, including 3,394,739 singleton term live births in Shandong Province, China, from January 1, 2016, to December 31, 2022. We used high-resolution spatiotemporal models based on residential addresses for exposure assessment. In addition to term LBW, we examined term small for gestational age (SGA) to capture fetal growth restriction while accounting for gestational age at birth. Given the low prevalence of these outcomes, we used logistic regression models where odds ratios approximated relative risks. A 4-step mediation analysis using logistic regression was conducted, followed by a counterfactual-based causal mediation analysis, to test the mediating role of gestational hypertension.

**Results:**

The mean (SD) O_3_ concentration was 113.90 (13.03) μg m^–3^. Each IQR increase in O_3_ was positively associated with the risks of term LBW (relative risk 1.055, 95% CI 1.034-1.077) and term SGA (relative risk 1.037, 95% CI 1.026-1.048). Using the traditional approach, gestational hypertension mediated 19.94% of the risk for term LBW and 13.41% for term SGA. Under the counterfactual framework, the contribution rates were 38.82% (term LBW) and 19.96% (term SGA) when excluding exposure-mediator interaction, and 35.15% (term LBW) and 18.82% (term SGA) when accounting for such interaction.

**Conclusions:**

Our findings showed that gestational hypertension was a significant mediator of the association between O_3_ exposure and risks of term LBW. Consequently, a multitiered strategy—encompassing stricter air quality standards, integrating O_3_ risk education into routine prenatal care, and taking proactive measures to minimize personal exposure—is essential to prevent potential adverse impacts on developing fetuses and mothers.

## Introduction

Low birth weight (LBW) is one of the major markers of fetal growth and stands as a predominant factor contributing to neonatal mortality and morbidity [1-3]. LBW infants are about 20 times more likely to die than heavier infants. Additionally, LBW is associated with adverse consequences in later life, such as neurobehavioral issues and respiratory, cardiovascular, cerebrovascular, and metabolic diseases [4-7]. LBW, which brings about a considerable disease burden and high medical costs, has presented significant challenges to health care systems. Consequently, it has been incorporated into the Sustainable Development Goals [8]. Nevertheless, despite years of strenuous efforts, merely a slight reduction in the prevalence of LBW has been noted [9], and factors such as multifetal pregnancy and maternal malnutrition, while recognized contributors, cannot fully account for the persistent prevalence of fetal growth restriction leading to LBW.

In recent years, regions with a high prevalence of LBW often face severe air pollution. A series of investigations noted that exposure to air pollution, such as particulate matter (PM), could plausibly be involved in LBW via higher oxidative stress levels [10,11]. Compared to PM, ozone (O_3_) is a pollutant endowed with more potent oxidative stress-inducing properties and is more challenging to mitigate through individual behavioral measures [12]. Thus, it is sensible to postulate that O_3_ exposure might potentiate susceptibility to LBW. However, little is known about the association between O_3_ exposure during pregnancy and the risk of LBW. Five studies have reported a positive and significant association between O_3_ exposure and LBW [13-17], whereas findings from another 5 studies were inconsistent or mixed [18-22]. Exposure misclassification might, to some extent, account for the variability in the results, which stems from regionally specific evaluations of pollution exposure [13-15,20,22]. Several recent studies further used satellite retrieval data to attenuate exposure misclassification to a certain degree [16,18,19,21]. However, most of these studies failed to account for several fundamental covariates such as gestational temperature exposure, maternal BMI, and the smoking status of the husband, which may lead to biased results [13,14,16,18-21]. Thus, more information may be needed in future studies.

Another pressing question is through which mechanisms O_3_ exposure during pregnancy affects fetal development in utero. Unlike acute and abruptly occurring conditions, the influence of O_3_ on fetal development unfolds gradually throughout pregnancy. In this chronic process, O_3_ can slow the development of the early placental vasculature and ultimately may promote maternal cardiovascular abnormalities later in gestation or intrauterine growth deficiency [23]. A wide range of studies has demonstrated that air pollution exposure could be involved in the development of hypertension through oxidative stress [24,25]. Three empirical studies focusing on the relationship between O_3_ exposure and the risk of gestational hypertension achieved similar results [26-28]. Gestational hypertension is evidenced by a reduction in placental perfusion [29,30], and the occurrence of intravascular coagulation [31,32], which is considered to precipitate placental dysfunction and subsequently lead to LBW [33]. Given the pairwise associations, we propose that gestational hypertension may mediate the associations between O_3_ and the risks of LBW and small for gestational age (SGA), which has yet to be studied.

To address the above gaps, our study estimated the independent effects of maternal O_3_ exposure on term LBW and SGA based on a broad and highly representative sample, while controlling for a range of covariates. Additionally, we examined, for the first time, the mediating effects of gestational hypertension to provide evidence regarding the underlying mechanisms.

## Methods

### Study Population

This is a retrospective cohort study based on the extended version of the Cheeloo Lifespan Electronic Health Research Data-library in Shandong Province, China (Multimedia Appendix 1). Shandong is a coastal province in eastern China (34°22.9′–38°24.01′N, 114°47.5′–122°42.3′E) with approximately 100 million inhabitants and substantial geographic and socioeconomic heterogeneity across coastal and inland areas. The foundation of our data infrastructure is the Shandong Province Birth Certificate System, a national government–administered registry that integrates birth information reported by medical institutions at multiple levels across the province. This system captures standardized demographic and administrative information for nearly all newborns and their parents, including place of residence (eg, municipality or township of residence). Additional data sources include linked electronic medical records (EMRs) and electronic health records, which provide complementary information on maternal health conditions, clinical encounters, and other relevant characteristics. A unique deidentified personal identification code is systematically applied across all databases; therefore, linkage of these records enables the construction of a large population-based birth cohort. During the study period, birth records were continuously available from 16 cities and 1840 township-level administrative units across the province.

We initially identified 7,141,641 live births recorded in Shandong Province between January 1, 2016, and December 31, 2022. Among these, 6,099,731 were singleton live births. Neonatal information was routinely verified by designated quality-control personnel at delivery institutions, and data underwent consistency and range checks, with obvious logic inconsistencies corrected when possible. To improve comparability and focus on a relatively homogeneous population, we restricted the cohort to singleton firstborn live births among women aged 20 to 45 years and further limited analyses to term births (gestational age 37‐45 wk) to reduce confounding related to prematurity, resulting in a total of 5,720,445 eligible births. We excluded records with incomplete hospitalization records or residential address information (n=2,130,147), unmarried women (n=125,465), missing O_3_ exposure (n=70,066), and implausible birthweight values (n=28). The final analytical sample included 3,394,739 births (Multimedia Appendix 2).

### Term LBW and SGA

Our key interests were term LBW and SGA. Term LBW was defined as birth weight less than 2500 g and gestational age greater than 37 weeks. In addition, term SGA was used in the analysis because it takes gestational length into consideration. Term SGA was determined as birth weight below the 10th percentile and gestational age greater than 37 weeks according to the INTERGROWTH-21st (International Fetal and Newborn Growth Consortium for the 21st Century) fetal growth standards [34].

### O_3_ Exposure

Daily concentrations of O_3_, with a spatial resolution of 1 km × 1 km, were sourced from the CHAP (China High Air Pollutants) dataset. The ground-level 8-hour maximum daily average O_3_ concentration was predicted through an ensemble learning approach using the space-time extremely randomized trees model, which incorporated solar radiation intensity, surface temperature, remote sensing products, and ground-based observations. The 10-fold cross-validation results for O_3_ demonstrated a coefficient of determination (*R*²) of 0.87 and a root mean square error of 17.10. Detailed information about the prediction is available elsewhere [35]. Address information for each participant was gathered and geocoded into longitude and latitude coordinates. We calculated the O_3_ exposure levels throughout the entire pregnancy for each subject based on their home address, the date of their last menstrual period, and the date of delivery. We also calculated pregnancy exposure to PM≤2.5 μm in aerodynamic diameter (PM_2.5_), PM≤10 μm in aerodynamic diameter (PM_10_), nitrogen dioxide (NO_2_), sulfur dioxide (SO_2_), and carbon monoxide (CO) concentrations using similar methods.

### Gestational Hypertension

Gestational hypertension was identified based on the *International Classification of Diseases, Tenth Revision* (ICD-10) [36] codes documented in integrated outpatient and inpatient medical records throughout pregnancy, which were derived from EMR. Specifically, the diagnosis was determined using ICD-10 diagnostic codes O13 (gestational [pregnancy-induced] hypertension), O14 (pre-eclampsia), and O15 (eclampsia), as well as the keyword “hypertensive disorders of pregnancy.” In addition, as a nationally implemented program, China’s National Essential Public Health Services includes standardized maternal follow-up management, during which pregnant women are routinely assessed for pregnancy complications such as gestational hypertension. This framework may support more complete case ascertainment across health care settings, including resource-limited areas, and help mitigate potential under-ascertainment and information loss.

### Covariates

Using a directed acyclic graph (Multimedia Appendix 3) informed by the literature [37], we selected confounders that are associated with both O_3_ exposure and birth outcomes and that, when adjusted for, remove all backdoor paths between the exposure and the outcome while minimizing overadjustment [38]. The models were adjusted for maternal age, maternal occupation (such as farmer, clerk, housewife, worker, and other categories), infant sex, gestational diabetes, and the smoking status of the husband (the prevalence of active smoking among pregnant women in China is exceptionally low) [13,18]. In addition, given the close association between temperature and O_3_ concentration, temperature exposure during pregnancy was included. Monthly ambient temperature was obtained from the Global Surface Summary of the Month data files provided by the National Oceanic and Atmospheric Administration (NOAA), with a spatial resolution of 10 km × 10 km [39].

### Ethical Considerations

This study was conducted in accordance with the Declaration of Helsinki and was approved by the ethics committee of the Shandong University School of Public Health (LL20240334). The requirement for informed consent was waived because all data were anonymized.

### Statistical Analysis

We used logistic regression models to estimate the associations of an IQR increase in O_3_ concentration with term LBW and SGA. Given the low prevalence of these outcomes in our study population (typically <10%), the odds ratios (ORs) derived from logistic regression were considered reasonable approximations of relative risks (RRs) [40]. Consequently, we reported the results as RRs and 95% CIs. To ensure the robustness of our findings, 3 sensitivity analyses were conducted. First, Cox proportional hazards models were fitted using gestational age (wk) as the time scale to incorporate time-to-event data. Second, 2-pollutant models were established by adjusting for PM_2.5_, PM_10_, NO_2_, SO_2_, and CO to isolate the independent effect of O_3_. Third, maternal BMI was added to control for nutritional status; missing BMI data were imputed using the “*missForest*” algorithm prior to analysis (Multimedia Appendix 4). Fourth, to address potential biases arising from (1) incomplete residential addresses or failed hospitalization record linkages and (2) nonrandom missingness of O_3_ exposure data, and to improve the representativeness of the analytic sample, we used a weighted logistic regression model supplemented by robust variance estimation. We first derived calibration weights for records with complete residential address and hospitalization information. These weights were constructed by calibrating the distribution of key demographic variables (eg, maternal age, infant sex, and gestational age) in our complete-case sample to the eligible population, thereby adjusting for potential bias due to missingness in these essential identifiers [41]. Next, to account for nonrandom missingness in O_3_ exposure assessment (eg, dependent on geographic location), we estimated inverse probability of selection weights (IPSWs) [42]. The IPSWs were derived from a logistic regression model predicting inclusion in the exposure-assessed analytic sample based on maternal age, infant sex, gestational diabetes, the smoking status of the husband, and maternal occupation. The final analytic weight for each participant was obtained by multiplying the calibration weight and the IPSW. To ensure numerical stability and facilitate variance estimation, the composite weights were normalized to sum to the actual sample size (ie, mean weight=1). Additional details are provided in Multimedia Appendix 5.

Stratified analyses were conducted based on (1) maternal age (<30 y vs ≥30 y, with the latter marking the initial decline in fecundity and ovarian function [43]; (2) infant sex (female vs male) [44]; and (3) coastal residence (coastal vs inland) to account for geographic environmental differences [45,46].

The hypothesis that the association of O_3_ exposure with term LBW and SGA would be mediated by gestational hypertension was tested using a 4-step analysis. The method involved testing the total effect (TE) of O_3_ exposure with term LBW and SGA and then estimating how much the association was reduced by the inclusion of gestational hypertension. Using a logistic regression model, the TE, represented as the RR of term LBW or SGA linked to an IQR rise in O_3_ exposure, was divided into direct and indirect effects in line with the methodology described by Buis [47]. The mediating role of gestational hypertension was quantified and evaluated within the framework of a Cox proportional hazards model.

To gain a deeper understanding of the underlying causal processes, a mediation approach based on the counterfactual framework for causal inference was adopted. In contrast to traditional methods, this framework defines mediation effects using counterfactual scenarios. This enables a more precise exploration of how the exposure and the potential mediator jointly affect the outcome [48,49]. In this counterfactual framework, the TE was defined as the RR of term LBW or SGA related to an IQR increase in O_3_ exposure, comparing the 75th percentile to the 25th percentile. This was calculated while adjusting for covariates and allowing gestational hypertension to occur naturally. Following Pearl’s [50] approach for the risk difference scale, the TE was expressed as the product of the ORs for the natural direct effect and the natural indirect effect (NIE) [49]. The natural direct effect was estimated by comparing O_3_ exposure at the 75th percentile versus the 25th percentile, while keeping gestational hypertension at the level it would have been under the 25th percentile of O_3_ exposure. The NIE was defined as the change in odds resulting from gestational hypertension when O_3_ exposure increased by an IQR. It represents the part of the causal pathway from exposure to outcome that was mediated by gestational hypertension. The proportion mediated was computed as log(NIE)/log(TE), following the method of Imai et al [51]. The details of the analysis are presented in Multimedia Appendix 6.

All statistical analyses were conducted using R (version 4.4.0; R Foundation for Statistical Computing) and Stata (version 17.0, StataCorp LLC). Bias-corrected standard errors and 95% CIs were estimated using bootstrapping with 1000 replications. All statistical tests were 2-sided, with a significance level set at *α*=.05.

## Results

### Descriptive Results

Table 1 presents the descriptive characteristics of the study population. Among the 3,394,739 term births included in this study, 1,615,993 (47.6%) were female. The overall prevalence of term LBW and SGA infants was 0.89% and 3.34%, respectively. Notably, a higher incidence of both term LBW and SGA was observed among mothers diagnosed with gestational hypertension. Regarding the air pollution levels during the study period, the summary statistics are shown in Table 2. The mean (SD) O_3_ concentration during pregnancy was 113.90 (13.03) µg m^–3^. Furthermore, O_3_ exposure was correlated with other pollutants, with the Pearson correlation coefficients ranging from –0.47 to 0.75.

**Table 1.** Characteristics of 3,394,739 singleton term live births included in the retrospective birth cohort study in Shandong Province, China, 2016‐2022 (N=3,394,739).

Characteristics	Total (N=3,394,739)	Term LBW^a^ (n=30,145)	Term SGA^b^^c^ (n=113,382)
Infant sex, n (%)			
Male	1,778,746 (52.4)	12,141 (40.3)	56,316 (49.7)
Female	1,615,993 (47.6)	18,004 (59.7)	57,066 (50.3)
Gestational age (wk), mean (SD)	39.0 (1.1)	38.0 (1.0)	39.1 (1.1)
Maternal age (y), mean (SD)	30.0 (4.8)	29.9 (5.3)	29.0 (5.0)
Maternal occupation, n (%)			
Clerk	563,149 (16.6)	4670 (15.5)	18,194 (16)
Farmer	1,121,893 (33)	10,371 (34.4)	39,293 (34.7)
Housewife	372,462 (11)	3682 (12.2)	12,951 (11.4)
Worker	220,077 (6.5)	1846 (6.1)	6713 (5.9)
Others	1,117,158 (32.9)	9576 (31.8)	36,231 (32)
Status of husband smoking, n (%)			
Yes	116,956 (3.4)	1123 (3.7)	3921 (3.5)
No	3,277,783 (96.6)	29,022 (96.3)	109,461 (96.5)
Maternal BMI (kg/m^2^), mean (SD)^d^	22.4 (4.0)	22.0 (4.1)	21.6 (3.8)
Gestational diabetes, n (%)			
Yes	349,910 (10.3)	2489 (8.3)	7850 (6.9)
No	3,044,829 (89.7)	27,656 (91.7)	105,532 (93.1)
Gestational hypertension, n (%)			
Yes	103,443 (3.1)	4915 (16.3)	8315 (7.3)
No	3,291,296 (96.9)	25,230 (83.7)	105,067 (92.7)

a LBW: low birth weight.

b SGA: small for gestational age.

c The sample size of term SGA was 3,394,049.

d The sample size of maternal BMI was 1,556,480.

**Table 2.** Distribution and correlation of air pollutant concentrations and temperature during pregnancy among the study population in Shandong Province, China (2016‐2022).

Exposure	Mean (SD)	Min-Max	Median (IQR),	O_3_^a^	PM_2.5_^a^^,^^b^	PM_10_^a^^,^^c^	NO_2_^a^^,^^d^	CO^a^^,^^e^	SO_2_^a^^,^^f^	Temperature^a^
O_3_ (μg/m^3^)	113.90 (13.03)	62.35-157.58	112.70 (103.60-23.53)	1.00	–0.47	–0.36	–0.42	–0.42	–0.39	0.75
PM_2.5_ (μg/m^3^)	53.01 (12.37)	17.76-111.27	52.80 (44.22-61.11)	—^g^	1.00	0.96	0.80	0.81	0.73	–0.35
PM_10_ (μg/m^3^)	97.25 (19.58)	35.03-191.31	97.37 (83.29-111.10)		—	1.00	0.81	0.77	0.72	–0.26
NO_2_ (μg/m^3^)	34.36 (6.37)	8.81-68.55	34.61 (30.04-38.93)			—	1.00	0.75	0.62	–0.43
CO (mg/m^3^)	0.98 (0.23)	0.44-2.55	0.94 (0.82-1.09)				—	1.00	0.81	–0.28
SO_2_ (μg/m^3^)	18.98 (8.94)	5.60-92.77	15.97 (12.36-23.45)					—	1.00	–0.14
Temperature (℃)	14.58 (3.06)	2.04-22.12	14.48 (12.09-17.22)						—	1.00

a These are *r* values with *P*<.001.

b PM_2.5_: particulate matter with a diameter of 2.5 μm or smaller.

c PM_10_: particulate matter with diameter of 10 μm or smaller.

d NO_2_: nitrogen dioxide.

e CO: carbon monoxide.

f SO_2_: sulfur dioxide.

g Not applicable.

### Association Between O_3_ Exposure and Term LBW or SGA

Table 3 illustrates the associations of O_3_ exposure with term LBW and SGA across various adjusted models. Based on logistic regression models, each IQR increase in O_3_ concentration was significantly associated with elevated risks of term LBW (RR 1.055, 95% CI 1.034-1.077) and SGA (RR 1.037, 95% CI: 1.026-1.048). The median (IQR) O_3_ concentration was 112.70 (19.93) μg/m^3^. These results were corroborated by Cox proportional hazards models, yielding hazard ratios of 1.071 (95% CI 1.049-1.093) and 1.081 (95% CI 1.069-1.092) for term LBW and SGA, respectively. Sensitivity analyses adjusting for maternal BMI (original and imputed) and co-pollutants confirmed the robustness of our findings. The weighted model produced RRs consistent with the main model (term LBW: RR 1.055, 95% CI 1.032‐1.077; term SGA: RR 1.033, 95% CI 1.022‐1.044). Additionally, stratified analyses showed stable positive associations across subgroups of maternal age, infant sex, and coastal residence (Multimedia Appendix 7).

**Table 3.** Associations between maternal ozone exposure and the risks of term low birth weight (LBW) and term small for gestational age (SGA) among singleton term births in Shandong Province, China, 2016‐2022^a^.

Model	RR^b^ (95% CI)	
	Term LBW (n=3,394,739)	Term SGA (n=3,394,049)
Main model^c^	1.055 (1.034-1.077)	1.037 (1.026-1.048)
Cox model^d^	1.071 (1.049-1.093)	1.081 (1.069-1.092)
Main model adjusted for maternal BMI^e^	1.051 (1.021-1.082)	1.033 (1.018-1.049)
Main model adjusted for imputed maternal BMI^f^	1.056 (1.034-1.078)	1.040 (1.029-1.051)
Main model adjusted for PM_2.5_^g^	1.058 (1.034-1.082)	1.072 (1.060-1.085)
Main model adjusted for PM_10_^h^	1.051 (1.028-1.074)	1.063 (1.051-1.075)
Main model adjusted for NO_2_^i^	1.056 (1.033-1.079)	1.048 (1.036-1.060)
Main model adjusted for SO_2_^j^	1.040 (1.017-1.063)	1.047 (1.035-1.059)
Main model adjusted for CO^k^	1.061 (1.038-1.085)	1.057 (1.045-1.069)
Main model adjusted for gestational hypertension	1.034 (1.013-1.055)	1.029 (1.019-1.040)
Weighted model ^l^	1.055 (1.032-1.076)	1.033 (1.022-1.044)

a Effect estimates were expressed per IQR increase in O_3_. The median (IQR) O_3_ concentration was 112.70 (19.93) μg/m³.

b RR: relative risk.

c Main model: single-pollutant model for O_3_ adjusted for maternal age, infant sex, temperature, maternal occupation, gestational diabetes, and the smoking status of the husband.

d Cox proportional hazards model (time scale: gestational age): adjusted for maternal age, infant sex, temperature, maternal occupation, gestational diabetes, and smoking status of the husband. Results are presented as hazard ratios (HRs) with 95% CIs.

e The sample size of maternal BMI was 1,556,480.

f Missing values were imputed using the missForest algorithm.

g PM_2.5_: fine particulate matter with a diameter of 2.5 μm or smaller.

h PM_10_: particulate matter with a diameter of 10 μm or smaller.

i NO_2_: nitrogen dioxide.

j SO_2_: sulfur dioxide.

k CO: carbon monoxide.

l Adjusted for maternal age, infant sex, gestational diabetes, maternal occupation, smoking status of the husband, and temperature category. Final weights were calculated as ranking weights multiplied by inverse probability of selection weight (IPSW) truncated at the 99th percentile.

Notably, while O_3_ exposure remained significantly associated with adverse outcomes after adjusting for gestational hypertension, the effect estimates were slightly attenuated (term LBW: RR 1.034, 95% CI 1.013-1.055; term SGA: RR 1.029, 95% CI 1.019-1.040), suggesting a potential mediating pathway, as shown in Table 4.

**Table 4.** Mediation of gestational hypertension in the association between ozone exposure and term low birth weight (LBW) and term small for gestational age (SGA) in the Shandong birth cohort (2016‐2022).

Mediation of gestational hypertension	Term LBW	Term SGA
Four-step analysis with the Sobel approach^a^		
Total effect, RR^b^ (95% CI)	1.042 (1.021-1.064)	1.034 (1.023-1.045)
Direct effect, RR (95% CI)	1.034 (1.013-1.055)	1.029 (1.019-1.040)
Indirect effect, RR (95% CI)	1.008 (1.008-1.009)	1.005 (1.004-1.005)
Mediation effect (%)	19.94	13.41
Causal mediation^a^ (excluding exposure-mediator interaction)		
Marginal total effect, RR (95% CI)	1.055 (1.034-1.077)	1.037 (1.026-1.048)
Natural direct effect, RR (95% CI)	1.034 (1.013-1.055)	1.029 (1.019-1.040)
Natural indirect effect, RR (95% CI)	1.021 (1.020-1.021)	1.007 (1.007-1.008)
Mediation effect (%)	38.82	19.96
Causal mediation^a^ (including exposure-mediator interaction)		
Marginal total effect, RR (95% CI)	1.055 (1.033-1.076)	1.036 (1.025-1.047)
Natural direct effect, RR (95% CI)	1.035 (1.014-1.056)	1.029 (1.019-1.040)
Natural indirect effect, RR (95% CI)	1.019 (1.018-1.021)	1.007 (1.006-1.007)
Mediation effect (%)	35.15	18.82

a The use of the bootstrap was proposed to estimate standard errors.

b RR: relative risk.

### The Mediation of Gestational Hypertension

Exposure to O_3_ was correlated with an elevated risk of developing gestational hypertension, with an OR of 1.162 (95% CI 1.149-1.175). Additionally, individuals who experienced gestational hypertension were more likely to deliver babies with term LBW (RR 6.499, 95% CI 6.300-6.706) and term SGA (RR 2.767, 95% CI 2.703,2.832; Multimedia Appendix 8). Table 4 presents the indirect contribution of gestational hypertension in the association between O_3_ exposure and term LBW or SGA. We found that gestational hypertension partly mediated these associations. Gestational hypertension accounted for 19.94% and 13.41% of the association between O_3_ exposure and term LBW or SGA, respectively. Under the framework of Cox proportional hazards models, consistent results were observed, with mediation proportions of 31.28% for term LBW and 11.35% for term SGA (Multimedia Appendix 9).

Under the counterfactual framework, the contribution of gestational hypertension to the association between prenatal O_3_ exposure and term LBW was 38.82% when excluding exposure-mediator interaction, and 35.15% when accounting for such interaction. For term SGA, the corresponding contribution rates were 19.96% (without interaction) and 18.82% (with interaction), respectively.

## Discussion

### Principal Findings

To our knowledge, this is the first study to investigate the mediation effects of gestational hypertension in the associations of O_3_ exposure during pregnancy with term LBW and SGA. Based on a birth cohort of 3,394,739 samples in China, this study provides novel findings by demonstrating the independent effect of O_3_ exposure on term LBW and SGA. Additionally, mediation analysis revealed that the proportion of the effect of O_3_ exposure on term LBW and SGA mediated through gestational hypertension ranges from 13.41% to 19.94%. This study holds significant implications for the surveillance and management of maternal blood pressure during prenatal care and contributes to our comprehension of the underlying mechanisms regarding the associations of O_3_ exposure with term LBW and SGA.

### Associations Between Prenatal O_3_ Exposure and Term LBW

Previous studies have revealed that PM exposure might impact fetal growth through elevated oxidative stress levels [10,11]. As another major ambient air pollutant, O_3_, with more powerful oxidative stress-eliciting characteristics [12], may act indirectly on the fetus by inducing oxidative stress, inflammatory responses, and hormonal imbalances in the mother [52]. These maternal states, over time, may disrupt normal placental function and fetal development, ultimately resulting in LBW [23]. Our study indicated a similarly notable association between O_3_ exposure during pregnancy and term LBW as well as term SGA. This is consistent with nationwide studies conducted in the United States with 2,179,040 participants and Iran with 4,030,383 participants [13,16]. However, some recent studies have reported conflicting findings [18-22], which may be attributed to differences in study design and the statistical models used.

It is well-known that variability in exposure assessment approaches is an important source of variability in effect estimates between different studies. Unlike previous studies that estimated exposure based on data from fixed monitoring stations, we used O_3_ concentrations retrieved from satellite remote sensing to calculate individual O_3_ exposure levels throughout pregnancy. This approach enables a more reliable estimation of the impact of O_3_ exposure on adverse birth outcomes. Additionally, by leveraging enriched indicators from EMRs and electronic health records, this study incorporated maternal and paternal information into the analytical models based on an extensive sample covering the period from 2016 to 2022. These factors have been demonstrated to potentially confound the associations under investigation [53]. A series of robust analyses further validates the reliability and significance of our results. Our findings provide justification for addressing air pollution-related adverse birth risk to achieve Sustainable Development Goals.

### Gestational Hypertension Mediated Partly the Associations Between Prenatal O_3_ Exposure and Term LBW

Fetal growth and development in utero are a continuous process, indicating that the impact of air pollution during pregnancy is likely to be long-term and chronic. Consequently, pregnancy complications and the associated physiological changes in the mother may play a crucial role in mediating these effects. Several studies have used effect modification to explore the mechanisms by which air pollution exposure affects birth outcomes, although the process through which air pollution exposure causes LBW remains elusive. Recent studies, including ours, have suggested that O_3_ or PM_2.5_ exposure in the first trimester is associated with gestational hypertension [26-28,54]. There is also evidence that gestational hypertension increases the risk of LBW [55,56]. Exposure to O_3_ may not only contribute to gestational hypertension but may also generate intergenerational effects, resulting in term LBW and SGA. However, none of these studies have explored whether gestational hypertension could link O_3_ exposure to adverse birth outcomes.

This present study found that gestational hypertension significantly mediated 13.41% and 19.94% of the association between O_3_ exposure and term LBW or SGA, respectively. These findings provide strong evidence that gestational hypertension occurring in later pregnancy, along with associated placental perfusion abnormalities, significantly mediates the effect of O_3_ exposure on fetal growth restriction. After entering the body, O_3_ primarily affects the respiratory and circulatory systems, where it induces oxidative stress and disrupts vascular tone regulation [57]. Mechanistically, O_3_-related oxidative stress and inflammation may impair endothelial function and activate vasoactive pathways, including the renin-angiotensin system (eg, angiotensin-converting enzyme/angiotensin II) and endothelin-1 signaling, thereby contributing to elevated blood pressure [58]. These changes may provoke enhanced sympathetic activity, thereby augmenting the risk of gestational hypertension [59]. Supporting evidence from pregnant animal models shows that mid-gestational O_3_ exposure can induce a mild but significant increase in maternal arterial blood pressure [57]. Gestational hypertension may adversely affect placental blood supply, leading to suboptimal perfusion [31,32,60]. This impairment restricts the delivery of oxygen and nutrients to the fetus [30]. Ultimately, such deprivation significantly increases the risk of low birth weight. In addition, experimental studies indicate that prenatal O_3_ exposure can increase circulating soluble fms-like tyrosine kinase-1 and elevate uterine artery resistance, accompanied by placental transcriptional changes consistent with preeclampsia-related pathways, providing biological plausibility for placental vascular maladaptation [61]. Beyond circulatory effects, O_3_ exposure may contribute to fetal growth disturbances through maternal chronic inflammation. Some inflammatory mediators, including interleukin-17A, have been shown to cross the placental barrier and potentially influence fetal developmental regulation [62].

To further quantify this mediation pathway, causal mediation analysis was conducted. When allowing for exposure-mediator interaction, the mediation effects were 35.15% for term LBW and 18.82% for term SGA. These findings suggest that, beyond acting through independent primary pathways, O_3_ exposure and gestational hypertension may also exert synergistic effects that exacerbate intrauterine fetal development impairments. Moreover, the mechanisms underlying these associations appear to be more complex than previously understood. Taken together, interventions or policies addressing O_3_ exposure should coordinate the health risks for mothers and neonates. Efforts to reduce the risks of LBW should be tailored to focus on mothers with gestational hypertension, while recognizing early warning signs of O_3_ exposure.

Collectively, we propose a multitiered strategy to mitigate the adverse effects of O_3_ exposure. At the policy level, stricter enforcement of air quality standards and the development of pregnancy-specific health warning systems are essential. Within the health care system, O_3_ risk education should be integrated into routine prenatal care, particularly for high-risk populations. At the individual level, we recommend that pregnant women monitor air quality indices, minimize outdoor activities during peak pollution, use indoor air purifiers, and maintain an antioxidant-rich diet. Synergistic efforts across these sectors are required to effectively protect maternal and fetal health.

### Contributions and Limitations

This study enriches the growing body of literature in 2 aspects. First, it evaluates the associations between O_3_ exposure and the health risks faced by mothers and neonates, thereby enhancing understanding of the intergenerational impacts. Second, it reveals the mediating role of gestational hypertension within the aforementioned association, aiming to comprehensively illustrate the mechanisms underlying these associations.

However, our study has several limitations. First, exposure misclassification was a potential issue in our research. Specifically, using a single address to estimate O_3_ exposure may not accurately reflect personal exposure, as individuals may commute to workplaces during the day. Due to data limitations, indoor air pollution was not accounted for. However, given the large sample size, the impact of exposure misclassification primarily manifests as a dilution of the study’s effect, making it unlikely to fundamentally reverse the association we observed. Second, gestational hypertension was ascertained using ICD-10 codes from pregnancy-related outpatient and inpatient EMRs, which may have under-captured milder or undiagnosed cases, particularly in rural or medically underserved settings. Such under-ascertainment could underestimate the prevalence of gestational hypertension and bias the estimated associations toward the null. Nevertheless, China’s National Essential Public Health Services include standardized maternal follow-up management, during which pregnant women are routinely assessed for conditions such as gestational hypertension, which may support more complete case ascertainment across health care settings. Future studies could link more granular clinical data (eg, serial blood pressure measurements and proteinuria indicators) to construct a more sensitive phenotyping definition, thereby validating and potentially refining the effect estimates reported here. Third, although covariates were selected a priori using a directed acyclic graph, residual confounding from unmeasured factors (eg, diet or other environmental exposures not available in our database) cannot be completely ruled out. Fourth, we recognize that the process of constructing the analytic sample may have introduced selection bias, potentially affecting the generalizability of our findings. Approximately 40% of eligible births were not included in the analytic sample, largely due to unsuccessful linkages to hospitalization records, incomplete residential addresses, and non-random missingness in O_3_ exposure data. Compared with the eligible population, the average maternal age in our analytic sample was slightly lower (29.85 y, SD 4.82 vs 30.16 y, SD 4.91), with a small standardized mean difference of 0.062. Infant sex was nearly identical (female 47.6% in the analytic sample vs 47.7% in the eligible population; standardized mean difference=0.003). The limited imbalance in measured age and sex distributions suggests our sample is representative. Additionally, evidence from prior hospital-based birth cohorts in China [63] suggests that excluding participants with missing address data or failed geocoding does not correlate with a higher risk of adverse birth outcomes. In summary, while selection bias cannot be entirely ruled out, its impact is likely minimal. To reduce the potential bias, a weighting analysis has been applied. Sensitivity analyses using weighting methods yielded consistent estimates, thereby reinforcing the validity and robustness of our primary conclusions. In future studies, we will refine the data collection process by strengthening multisource record linkages and improving the precision of residential history tracking to further enhance data completeness and minimize potential selection bias. Fifth, our study was restricted to Shandong Province, China; therefore, caution is warranted when extrapolating the findings to regions with different climatic conditions, demographic structures, health care access, or pollutant concentrations, which may yield heterogeneous associations. Shandong spans substantial east-west variation and shares several natural and socioeconomic characteristics with China overall, providing a reasonable basis for examining the health implications of O_3_ exposure. Subgroup analyses also support the robustness of this study. However, the exposure distribution in our cohort was dominated by relatively high ambient O_3_ levels. Accordingly, applying these estimates to areas with lower O_3_ concentrations should be done cautiously, as the underlying exposure distribution may differ from that of our study population.

### Conclusions

This study provides, for the first time, robust evidence quantifying gestational hypertension as a significant mediator between prenatal O_3_ exposure and adverse birth outcomes—19.94% for term LBW and 13.41% for term SGA. Using a counterfactual mediation framework, we clarify a key biological pathway linking air pollution to impaired fetal growth. These findings highlight the need to integrate O_3_ pollution control into prenatal health guidance and strengthen blood pressure monitoring during pregnancy. Future research should validate these associations across diverse populations and investigate underlying biological mechanisms, such as oxidative stress.

## Supplementary material

10.2196/81412 Multimedia Appendix 1 Map of the study site in Shandong Province, China.

10.2196/81412 Multimedia Appendix 2 Study flowchart for participant inclusion and exclusion.

10.2196/81412 Multimedia Appendix 3 Directed acyclic graph of the associations between ozone exposure and child weight.

10.2196/81412 Multimedia Appendix 4 Imputation procedure based on random forest.

10.2196/81412 Multimedia Appendix 5 Weighting approach.

10.2196/81412 Multimedia Appendix 6 A 4-step analysis to test the mediator of gestational hypertension.

10.2196/81412 Multimedia Appendix 7 Associations of ozone exposure with term low birth weight and term small for gestational age, stratified by maternal age, infant sex, and coastal residence.

10.2196/81412 Multimedia Appendix 8 Association of ozone exposure with gestational hypertension, term low birth weight, and term small for gestational age.

10.2196/81412 Multimedia Appendix 9 Mediation analysis of gestational hypertension on the association between ozone exposure and term low birth weight and term small for gestational age based on Cox proportional hazards models.

## Abbreviations

CHAP: China High Air Pollutants
EMR: electronic medical record
ICD-10: *International Classification of Diseases, Tenth Revision*
INTERGROWTH-21st: International Fetal and Newborn Growth Consortium for the 21st Century
IPSW: inverse probability of selection weight
LBW: low birth weight
NIE: natural indirect effect
NOAA: National Oceanic and Atmospheric Administration
OR: odds ratio
PM: particulate matter
RR: relative risk
SGA: small for gestational age
TE: total effect
