# Supplementary figures and images for: Gestational Hypertension as a Mediator of Prenatal Ozone Exposure and Term Low Birth Weight: Birth Cohort Study
Source: JMIR Public Health Surveill. 2026 Apr 8;12:e81412. doi: 10.2196/81412 (PMC13061370; doi:10.2196/81412)

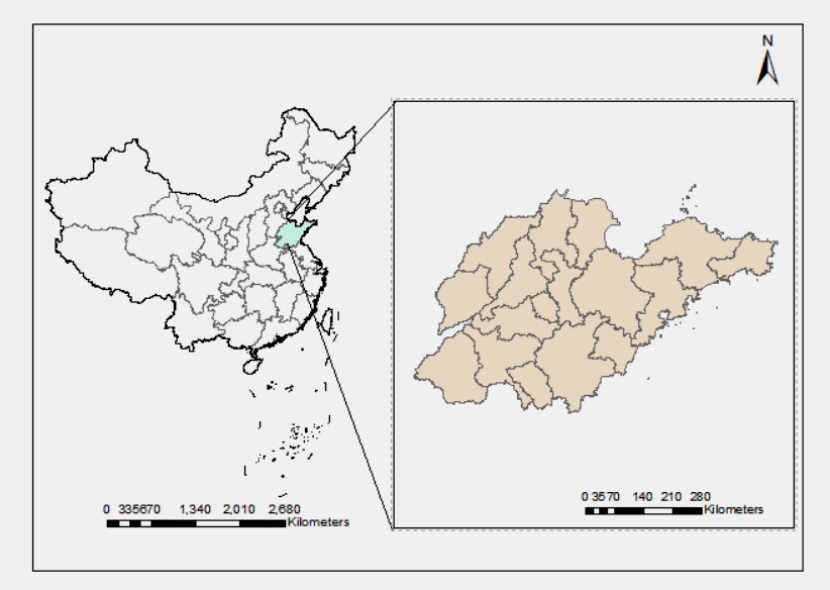

Supplement: Multimedia Appendix 1 [file publichealth-v12-e81412-s001.png]

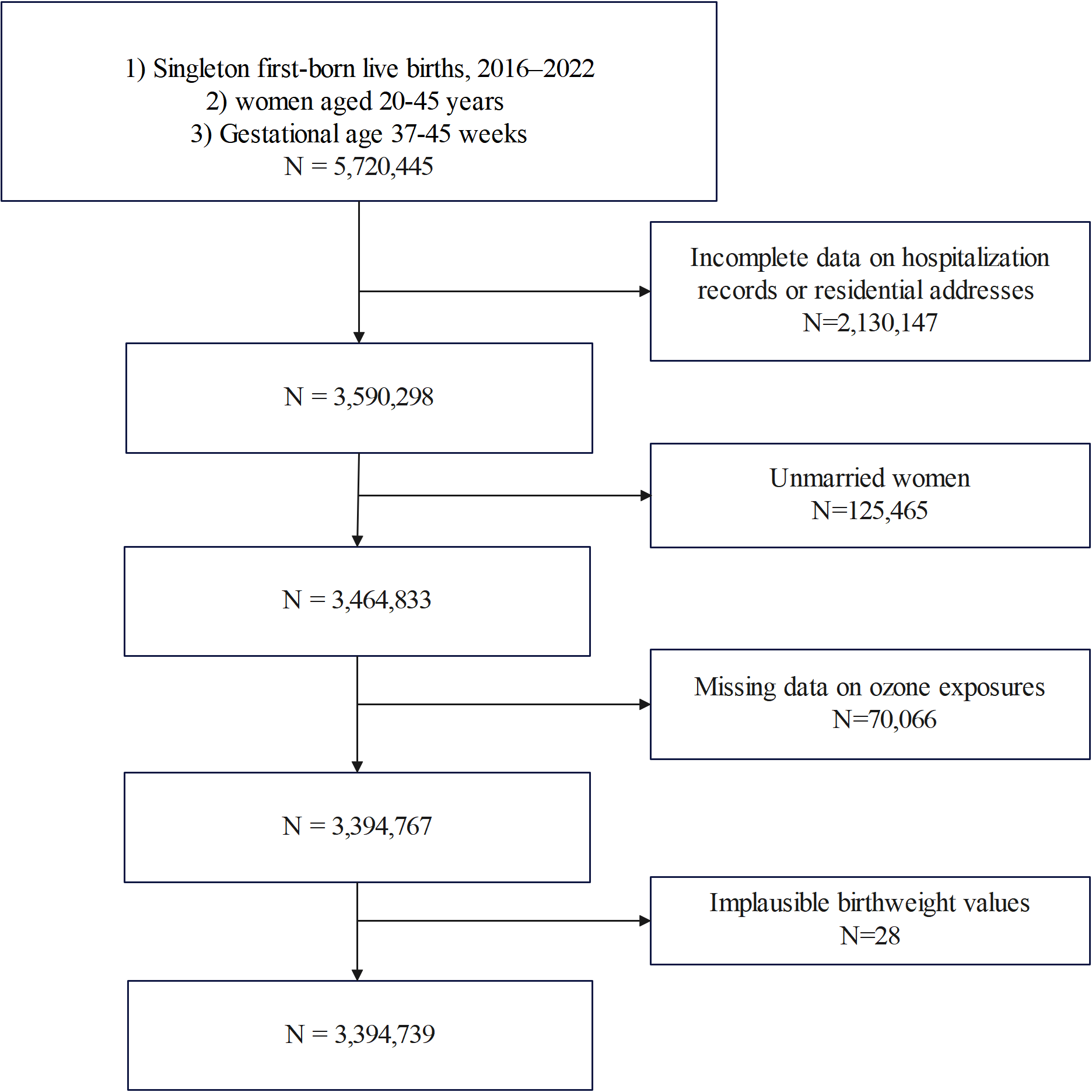

Supplement: Multimedia Appendix 2 [file publichealth-v12-e81412-s002.png]

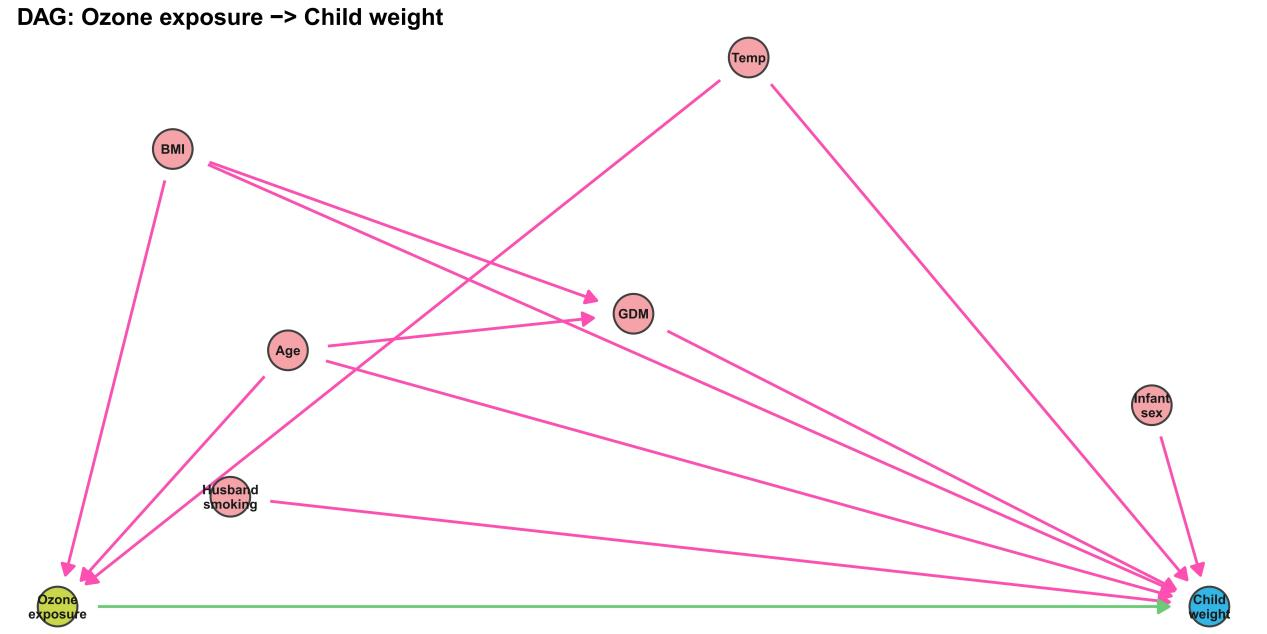

Supplement: Multimedia Appendix 3 [file publichealth-v12-e81412-s003.png]
