# Supplementary material for: Gestational Hypertension as a Mediator of Prenatal Ozone Exposure and Term Low Birth Weight: Birth Cohort Study
Source: JMIR Public Health Surveill. 2026 Apr 8;12:e81412. doi: 10.2196/81412 (PMC13061370; doi:10.2196/81412)
Supplement: Multimedia Appendix 4 [file publichealth-v12-e81412-s004.docx]

**Multimedia Appendix 4. Imputation procedure based on random forest.**

In this study, we used the missForest algorithm (R package: missForest) to impute missing values in maternal body mass index (BMI). The imputation model incorporated both continuous and categorical variables, including: ozone exposure, maternal age, maternal occupation (such as farmer, clerk, housewife, worker, and other categories), infant sex, ambient temperature category, gestational diabetes, and smoking status of husband. Because these variables are mixed-type and may relate to each other through nonlinear effects and interactions, a non-parametric tree-ensemble approach is appropriate for capturing complex dependencies without distributional assumptions. Traditional imputation methods, such as those based on multivariate normality assumptions (e.g., multiple imputation via chained equations, MICE), are less suitable in this context due to their limitations in handling categorical variables and nonlinear relationships. In contrast, missForest is a non-parametric, tree-based machine learning approach that has demonstrated superior performance in imputing mixed-type data, particularly in large epidemiological datasets[1, 2].

In our study, missingness was present in maternal BMI, and all other variables were used as predictors in the imputation model. The imputation proceeds as follows:

1.Initialization: Missing values in maternal BMI were initially filled with the mean of the observed maternal BMI values (the default missForest treatment for continuous variables), yielding a preliminarily completed dataset.

**2.Iterative model fitting (regression forest):** In each iteration, a random-forest regression model was trained using the individuals with observed maternal BMI, with the remaining variables as predictors. In our implementation, the number of trees was set to 200, offering a practical trade-off between computational cost and imputation accuracy. Each forest aggregates predictions from 200 independently grown decision trees, which improves stability and reduces variance in the imputed values.

3.Prediction and update: The fitted model was used to predict the missing maternal BMI values for individuals whose BMI was missing. Steps 2 and 3 were repeated until convergence.

This iterative refinement leverages the full variable set to progressively improve the imputed dataset until convergence.

**References**

[1] Stekhoven DJ, Buhlmann P: **MissForest--non-parametric missing value imputation for mixed-type data**. *Bioinformatics* 2012, **28**(1):112-118.DOI: 10.1093/bioinformatics/btr597.

[2] Tiwaskar S, Rashid M, Gokhale P: **Impact of machine learning-based imputation techniques on medical datasets- a comparative analysis**. *Multimedia Tools and Applications* 2024, **84**(9):5905-5925.DOI: 10.1007/s11042-024-19103-0.
