# Supplementary material for: Gestational Hypertension as a Mediator of Prenatal Ozone Exposure and Term Low Birth Weight: Birth Cohort Study
Source: JMIR Public Health Surveill. 2026 Apr 8;12:e81412. doi: 10.2196/81412 (PMC13061370; doi:10.2196/81412)
Supplement: Multimedia Appendix 5 [file publichealth-v12-e81412-s005.docx]

### **Multimedia Appendix 5. Weighting approach.**

To improve the representativeness of our analytic sample and address potential biases arising from (1) incomplete residential addresses or failed hospitalization record linkages, and (2) non-random missingness of ozone exposure data, we implemented a two-stage weighting procedure followed by a weighted sensitivity analysis.

**Stage 1: Calibration to the eligible population**

We derived calibration weights to align the distribution of key demographic characteristics in our sample with complete residential address and hospitalization information with those of the eligible birth population (N = 5,720,445), for which population-level margins were available from the provincial birth registry. Calibration is particularly advantageous in our setting because it does not rely on correctly specifying a parametric selection model, thereby avoiding potential bias from model misspecification. Moreover, it performs well even when only a limited number of auxiliary variables are available for adjustment—making it well-suited to our context where reliable population margins are restricted to a few key demographic characteristics [1].

*Step 1:* We selected maternal age, infant sex, and gestational age as calibration variables. These variables were retained because, as fundamental demographic stratifier, they are known associations with air pollution effects and are routinely used in birth cohort calibrations [2]. Maternal age was prioritized over paternal age due to its stronger empirical association with both exposure and outcome, and to avoid potential instability from high collinearity between parental ages (Spearman’s ρ = 0.85).

*Step 2 :* Calibration weights were computed using the *calibrate( )* function in the R *survey* package under the generalized regression framework, enforcing exact agreement between the weighted sample totals and population margins for maternal age, infant sex and gestational age. The solution minimized the entropy distance from base weights [1, 3, 4].

*Step 3:* Balance was assessed via standardized mean differences (SMDs). Post-calibration, SMDs for maternal age, infant sex and gestational age were <0.10, confirming close alignment with the source cohort (Multimedia Appendix 5a).

**Stage 2: Inverse Probability of Selection Weighting (IPSW) for exposure assessment**

Among individuals with complete address and hospitalization records, ozone exposure estimates were available for a subset. Given that this missingness is likely systematic (e.g., dependent on geographic location), we estimated IPSWs to adjust for potential bias in exposure assessment.

*Step 1:* A multivariable logistic regression model predicted inclusion in the exposure-assessed analytic sample. Following Metten et al., the IPSW model included predictors of both the outcome and exposure availability [5], including maternal age, infant sex, gestational diabetes, status of husband smoking and maternal occupation [6]. To examine potential effect modification, we tested the inclusion of interaction terms (e.g., maternal age × status of husband smoking), but found no significant improvement in model fit (likelihood ratio test, P > 0.05). Therefore, only main effects were retained for parsimony. Individuals with missing values in any predictor were excluded from this stage.

*Step 2:* The IPSW was defined as the inverse of the predicted inclusion probability and truncated at the 99th percentile to improve stability [7].

*Step 3:* Post-weighting balance was confirmed: all SMDs were <0.10 (Multimedia Appendix 5b), indicating adequate covariate balance.

**Final Weight Construction**

The final analytic weight for each participant was computed as the product of the Stage 1 calibration weight and the truncated Stage 2 IPSW:

$w_{final}=w_{rake}\times w_{IPSW,trunc}$.

These composite weights were normalized to sum to the final sample size (mean = 1) and applied in weighted logistic regression models as part of our sensitivity analysis, jointly addressing both distributional misalignment with the target population and selective missingness in exposure assessment.

**Multimedia Appendix 5a. Demographic characteristics of the sample with complete residential and hospitalization data before and after calibration weighting.**

| **Variable** | **Eligible population**  **N=5,720,445** | **Sample with complete residential address and hospitalization information**  **N=3,590,298** | | **SMD** |
| --- | --- | --- | --- | --- |
|  |  | **Unweighted sample** | **Weighted sample** |  |
| Maternal age (years, Mean (SD)) | 30.16 (4.91) | 29.93 (4.85) | 30.16 (4.88) | 0.00 |
| Female infants, n (%) | 2,731,240 (47.75) | 1,709,940 (47.63) | 2,731,240 (47.75) | 0.00 |
| Gestational age (weeks, Mean (SD)) | 39.04 (1.05) | 39.02 (1.06) | 39.04 (1.06) | 0.00 |

**Multimedia Appendix 5b. Characteristics of the exposure-assessed analytic sample before and after IPSW.**

| **Variable** | **Sample with complete residential address and hospitalization information**  **N=3,590,298** | **Exposure-assessed analytic sample** | | **SMD** |
| --- | --- | --- | --- | --- |
|  |  | **Unweighted sample**  **N=3,394,739** | **Weighted sample**  **N=3,590,298** |  |
| Maternal age (mean) | 29.93 (4.85) | 30.0 (4.80) | 29.94 (4.79) | 0.00 |
| Female infants, n (%) | 1,709,940 (47.63) | 1,615,993 (47.60) | 1,709,940 (47.63) | 0.00 |
| Gestational diabetes, n (%) | 369,751 (10.30) | 349,910(10.31) | 369,751 (10.30) | 0.00 |
| Status of husband smoking, n (%) | 121,214 (3.38) | 116,956 (3.45) | 121,214 (3.38) | 0.00 |
| Maternal occupation, n (%) |  |  |  |  |
| clerk | 595,630 (16.59) | 563,149 (16.59) | 595,630 (16.59) | 0.00 |
| farmer | 1,170,078 (32.59) | 1,121,893 (33.05) | 1,170,078 (32.59) | 0.00 |
| housewife | 407,858 (11.36) | 372,462 (10.97) | 407,858 (11.36) | 0.00 |
| others | 1,181,926 (32.92) | 1,117,158 (32.91) | 1,181,926 (32.92) | 0.00 |
| worker | 234,806 (6.54) | 220,077 (6.48) | 234,806 (6.54) | 0.00 |

**References**

[1] Deville J-C, Särndal C-E, Sautory O: **Generalized Raking Procedures in Survey Sampling**. *Journal of the American Statistical Association* 1993, **88**(423):1013-1020.DOI: 10.1080/01621459.1993.10476369.

[2] Wang Q, Miao H, Warren JL, Ren M, Benmarhnia T, Knibbs LD, Zhang H, Zhao Q, Huang C: **Association of maternal ozone exposure with term low birth weight and susceptible window identification**. *Environment International* 2021, **146**.DOI: 10.1016/j.envint.2020.106208.

[3] Lumley T, Shaw PA, Dai JY: **Connections between survey calibration estimators and semiparametric models for incomplete data**. *Int Stat Rev* 2011, **79**(2):200-220.DOI: 10.1111/j.1751-5823.2011.00138.x.

[4] Yap S, Luo Q, Wade S, Weber M, Banks E, Canfell K, O’Connell DL, Steinberg J: **Raking of data from a large Australian cohort study improves generalisability of estimates of prevalence of health and behaviour characteristics and cancer incidence**. *BMC Medical Research Methodology* 2022, **22**(1):140.DOI: 10.1186/s12874-022-01626-5.

[5] Metten MA, Costet N, Multigner L, Viel JF, Chauvet G: **Inverse probability weighting to handle attrition in cohort studies: some guidance and a call for caution**. *BMC Med Res Methodol* 2022, **22**(1):45.DOI: 10.1186/s12874-022-01533-9.

[6] Cole SR, Hernán MA: **Constructing Inverse Probability Weights for Marginal Structural Models**. *American Journal of Epidemiology* 2008, **168**(6):656-664.DOI: 10.1093/aje/kwn164.

[7] Austin PC, Stuart EA: **Moving towards best practice when using inverse probability of treatment weighting (IPTW) using the propensity score to estimate causal treatment effects in observational studies**. *Stat Med* 2015, **34**(28):3661-3679.DOI: 10.1002/sim.6607.
