# Supplementary material for: Gestational Hypertension as a Mediator of Prenatal Ozone Exposure and Term Low Birth Weight: Birth Cohort Study
Source: JMIR Public Health Surveill. 2026 Apr 8;12:e81412. doi: 10.2196/81412 (PMC13061370; doi:10.2196/81412)
Supplement: Multimedia Appendix 6 [file publichealth-v12-e81412-s006.docx]

**Multimedia Appendix 6. A 4-step analysis to test the mediator of gestational hypertension.**

The hypothesis that the association of ozone exposure with term low birth weight (LBW) and small for gestational age (SGA) will be mediated by gestational hypertension was tested using a four-step analysis with Sobel approach[1, 2]. Specifically, we tested the direct path of ozone exposure with term LBW and SGA, and estimated the extent to which the association was weakened by the inclusion of gestational hypertension. This analysis addresses the following questions:

*Step 1*: estimating the strength of the direct path. A Logistic model was constructed to test whether ozone exposure was significantly associated term LBW and SGA without adjusting for gestational hypertension.

$$P\left( H_{i}=1 \right)=c_{1}+c_{2}*{ozone}_{i}+c_{3}*{CON}_{i}$$

Where, $H_{i}=1$ indicates respondents having term LBW and SGA; ${ozone}_{i}$ measures ozone exposure, ${CON}_{i}$ are covariates. $c_{1}$, $c_{2}$, $c_{3}$, are the coefficients to measure. If ozone exposure was significantly associated term LBW or SGA, the mediator of gestational hypertension in the association was then tested.

*Step 2*: a Logistic model was applied to estimate whether ozone exposure was significantly related to gestational hypertension.

$$P\left( {gh}_{i}=1 \right)=a_{1}+a_{2}*{ozone}_{i}+a_{3}*{CON}_{i}$$

Where, ${gh}_{i}$ presents gestational hypertension, and $a_{1}$, $a_{2}$, $a_{3}$, are the coefficients to measure.

Collect the parameter estimate $a_{2}$, and its standard error, $S_{a}$.

*Step 3*: a Logistic model to estimate the association of ozone exposure with term LBW and SGA upon the addition of gestational hypertension to the model.

$$P\left( H_{i}=1 \right)=b_{1}+b_{2}*{ozone}_{i}+b_{3}*{CON}_{i}$$

Where, $b_{1}$, $b_{2}$, $b_{3}$ are the coefficients to measure.

Collect the parameter estimate $b_{2}$, and its standard error, $S_{b}$.

Using the coefficient estimates and and their standard errors and from *step 2* and *3*, compute the standardized elements: $a_{2}$, $b_{2}$ and their collected standard error $S_{a}$ and $S_{b}$ from step 2 and 3, compute the standardized elements: $Z_{a}=\frac{a_{2}}{s_{a}}$, $Z_{b}=\frac{b_{2}}{s_{b}}$, their product: $Z_{a*b}=Z_{a}*Z_{b}$, and their collected standard error $\sqrt{1+{{{Z_{a}}^{2}+Z}_{b}}^{2}}$

*Step 4*: compute the z-test that combines results from *step 2* and *3*, to indicate whether there is a significant mediation effect:

$$Z_{med}=\frac{Z_{a*b}}{\sqrt{1+{{{Z_{a}}^{2}+Z}_{b}}^{2}}}$$

Test $Z_{med}$ was significant at the p=0.05 level if it exceeds |1.96| (for a 2-tailed test with p=0.05), gestational hypertension was regarded as a mediator of the association between ozone and term LBW and SGA.

Then, following Buis, total effects were calculated in logistic regression, and the effects decomposed into direct and indirect effects[3]. The use of the bootstrap was proposed in order to estimate standard errors.

**References**

[1] Iacobucci D: **Mediation analysis and categorical variables: The final frontier**. *J Consum Psychol* 2012, **22**(4):582-594.DOI: 10.1016/j.jcps.2012.03.006.

[2] VanderWeele TJ: **Mediation Analysis: A Practitioner's Guide**. *Annu Rev Publ Health* 2016, **37**:17-32.DOI: 10.1146/annurev-publhealth-032315-021402.

[3] Buis ML: **Direct and indirect effects in a logit model**. *Stata Journal* 2010, **10**(1):11-29.DOI: Doi 10.1177/1536867x1001000104.
