# Supplementary material for: Gestational Hypertension as a Mediator of Prenatal Ozone Exposure and Term Low Birth Weight: Birth Cohort Study
Source: JMIR Public Health Surveill. 2026 Apr 8;12:e81412. doi: 10.2196/81412 (PMC13061370; doi:10.2196/81412)
Supplement: Multimedia Appendix 7 [file publichealth-v12-e81412-s007.docx]

|  |  | **Term LBW** | | **Term SGA** | |
| --- | --- | --- | --- | --- | --- |
|  |  | Number of women | RR (95% CI) | Number of women | RR (95% CI) |
| Maternal age | ＜30 years | 1,644,505 | 1.097 (1.066,1.129) | 1,644,092 | 1.080(1.065,1.095) |
|  | ≥30 years | 1,750,234 | 1.018 (0.989,1.048) | 1,749,957 | 1.004(0.988,1.020) |
| Infant sex | Male | 1,778,746 | 1.055 (1.021,1.089) | 1,778,458 | 1.052(1.019,1.087) |
|  | Female | 1,615,993 | 1.055(1.028,1.084) | 1,615,591 | 1.039(1.023,1.055) |
| Geographic location | Coastal | 1,091,629 | 1.118 (1.075,1.162) | 1,091,519 | 1.042(1.021,1.063) |
|  | Inland | 2,303,110 | 1.026(1.001,1.051) | 2,302,530 | 1.024(1.011,1.037) |

**Multimedia Appendix 7. Associations of ozone exposure with term low birth weight and term small for gestational age, stratified by maternal age, infant sex, and coastal residence.**

Abbreviations: term LBW, term low birth weight; SGA, term small for gestational age; RR, relative risk; CI, confidence interval.
