# Supplementary material for: Gestational Hypertension as a Mediator of Prenatal Ozone Exposure and Term Low Birth Weight: Birth Cohort Study
Source: JMIR Public Health Surveill. 2026 Apr 8;12:e81412. doi: 10.2196/81412 (PMC13061370; doi:10.2196/81412)
Supplement: Multimedia Appendix 8 [file publichealth-v12-e81412-s008.docx]

**Multimedia Appendix 7. Association of ozone exposure with gestational hypertension, term low birth weight and term small for gestational age.**

|  | **Effect size, RR (95% CI)** | | |
| --- | --- | --- | --- |
|  | Gestational hypertension | Term LBW | Term SGA |
| Ozone exposure | 1.162(1.149-1.175) |  |  |
| Gestational hypertension |  | 6.499(6.300-6.706) | 2.767(2.703-2.832) |

Models were adjusted for maternal age, infant sex, temperature, maternal occupation, gestational diabetes and smoking status of husband.

Abbreviations: term LBW, term low birth weight; SGA, term small for gestational age; RR, relative risk; CI, confidence interval.
