# Supplementary material for: Gestational Hypertension as a Mediator of Prenatal Ozone Exposure and Term Low Birth Weight: Birth Cohort Study
Source: JMIR Public Health Surveill. 2026 Apr 8;12:e81412. doi: 10.2196/81412 (PMC13061370; doi:10.2196/81412)
Supplement: Multimedia Appendix 9 [file publichealth-v12-e81412-s009.docx]

**Multimedia Appendix 8. Mediation analysis of gestational hypertension on the association between ozone exposure and term low birth weight and term small for gestational age based on Cox proportional hazards models.**

| **Mediation of gestational hypertension** | **Term LBW** | **Term SGA** |
| --- | --- | --- |
| **Mediation effect** | | |
| Total effect[HR (95% CI)] | 1.071(1.042,1.093) | 1.081 (1.070,1.090) |
| Direct effect[HR (95% CI)] | 1.048 (1.020,1.070) | 1.071 (1.050,1.081) |
| Indirect effect[HR (95% CI)] | 1.022 (1.020,1.023) | 1.009 (1.008,1.010) |
| Mediation effect percent(%) | 31.28 | 11.35 |

Models were adjusted for maternal age, infant sex, temperature, maternal occupation, gestational diabetes and smoking status of husband.

Abbreviations: term LBW, term low birth weight; SGA, term small for gestational age; HR, hazard ratio; CI, confidence interval.
